# Mechanisms for Migration of Alkali in Dolomitic Limestones

**DOI:** 10.3390/ma18184404

**Published:** 2025-09-21

**Authors:** Xinyu Zhang, Wei Li, Xiaojun Huang, Zhixin Wang, Min Deng

**Affiliations:** College of Materials Science and Engineering, Nanjing Tech University, Nanjing 211816, China

**Keywords:** alkali–dolomite reaction (ADR), alkali ion migration, microstructure, dedolomitization

## Abstract

The alkali–dolomite reaction (ADR) describes the interaction between alkalis in concrete and dolomite which results in dedolomitization, leading to cracking and deterioration of the concrete. A large number of research has explored the chemical products associated with the ADR, mechanisms of expansion, and methods of identification, but our understanding of the occurrence and progression of the ADR chemical reaction is substantially limited. Key factors controlling the ADR chemical reaction are generally not understood. This paper investigates the migration process of alkali ions in dolomitic limestone and reaction process with dolomite crystals and alkali. Dolomitic limestone samples were selected for experimentation. The amount of Sodium (Na^+^) was measured as a means of assessing alkali ion migration. We measured the degree of dedolomitization using X-ray diffraction (XRD). Microstructure was evaluated using field emission scanning electron microscopy (FESEM). This research provides new insights into dedolomitization. The pore network provides the physical pathway for alkali ion migration. Concentration gradients drive the migration of alkali ions, and their interactions control the efficiency of alkali ion migration.

## 1. Introduction

Alkali–dolomite reaction (ADR, frequently referred to as alkali–carbonate reaction (ACR)) is a chemical reaction that can lead to the expansion and cracking of concrete that involves alkalis and dolomitic rocks [1,2,3]. ADR was first documented by Swenson in the 1950s when he was observing severely cracked concrete made with a carbonate rock in Kingston, Canada [4]. The cracks were different from those in the concrete due to alkali–silica reaction (ASR). He stated that aggregates with carbonate-bearing components contribute significantly to the expansion and cracking of concrete. After this, the same type of damage was found in Indiana, South Dakota, and Illinois in the United States [5,6]. Based on an organized study of the rock samples from various regions of China by Deng Min et al., findings to date suggest that there are active mineral constituents which may lead to alkali–carbonate reaction (ACR), found in different rock types which are widely spread throughout China [7,8,9].

Since then, there has been an interest in ADR and the research community has taken an interest in other countries as well [6,10,11,12].

The alkali–dolomite reaction is shown in Equation (1):

CaMg(CO_3_)_2_ + 2MOH→Mg(OH)_2_ + CaCO_3_ + M_2_CO_3_ (1)

where M represents the monovalent alkali cation (Na^+^, K^+^).

The mechanism of expansion of ADR has always been a considerable focus and a challenge in research. A number of mechanisms of expansion have been proposed but some mechanisms have been gradually refuted over time. Two most cited theories documented in the literature include the theories of Tang et al. [13,14,15] and the theory of Katayama et al. [16,17,18,19]. The theory, originally proposed by Tang and Deng [13,14,15] is outlined as follows: (1) the reagents migrate inward through the argillaceous network and are driven by the Gibbs free energy of the reaction; and (2) brucite and calcite grow in the limited space of the dolomite crystals creating crystallization pressure and resulting in expansion of rock. Tang et al.’s mechanism of expansion has flaws. The role of ion migration in ADR is unclear. The changes in Gibbs free energy are key to dedolomitization. However, Gibbs free energy does not drive alkalizing ion migration within rocks. Katayama and Grattan-Bellew [16,17,18,19] argued that simple dedolomitization is not sufficient for causing concrete expansion and cracking. They posited that the primary cause is alkali–silica reaction (ASR) caused by microcrystalline quartz. The mechanism of expansion proposed by Katayama et al. has its limitations. It is possible to decrease ASR by including 20% fly ash and 35% slag cement as bulk replacement materials; however, these scenarios did not decrease ADR. Chen et al. proposed a new alkaline medium, tetramethylammonium hydroxide (TMAH), that would react only with dolomite and not quartz. In their studies, dolomitic rock still had continual expansion with TMAH solution immersion, which terminates the argument meditating that ADR would not have expansion [20]. Mao has also confirmed compacted dolomite has significant expansive stresses in an alkaline solution. The osmosis theory, initially proposed by Hadley, was later questioned and recently interpreted by Milanesi et al. [21]. They consider that carbonate ions (CO_3_^2−^) from reactions play a crucial role in ADR expansion caused by dedolomitization. Even though there is a body of international research on the chemical reaction products, expansion mechanisms, and identification methods of ADR [22,23,24,25,26,27,28], its occurrence and development process remain unclear.

Currently, only a few studies were focused on the ion migration in alkali reactive aggregate. In ASR, researchers have proposed that the formation of an ASR gel can only be present at reactive silica sites with alkali and hydroxy ions migrating to these sites. Additionally, the rate of migration mainly depends on the concentration gradient of each reaction site [7]. However, none of these speculations are based on the results of experimental research, and researchers have paid little attention to ion migration in alkali reactive aggregate. Li et al. [29] have made some progress in the migration process of OH^−^ ions in dolostone. In his study, OH^−^ ions react with dolomite grains and form a nanofiltration-like structure that may block the migration of alkali ions and inhibit the dedolomitization process. Duke [30] proposed that dedolomitization only occurs at the aggregate edges. Calcium and magnesium ions will not diffuse, but carbonate ions can move freely. However, the key controlling factors remain unclear, and the key parameters driving ADR activity in dolomitic limestone are not clearly identified.

Moreover, systematic petrological approaches have been widely used to evaluate the alkali–aggregate reaction (AAR) process [29,31,32]. The polarizing microscopy has been employed to identify reaction characteristics for active silica phases and dolomite. With development of advanced imaging capabilities such as field emission scanning electron microscopy (FESEM) and backscattered electron imaging (BSE), these studies have determined the microstructure of reaction boundaries and elemental distributions using energy dispersive spectroscopy. These methods have created a relevance among mineral composition, microstructure, and reactive activity. Attending to the common approaches detailed above, the present study uses polarized light microscopy and FESEM/BSE as the principle analytical tools to evaluate the migration behavior of alkali ions within dolomitic limestone and examine reactions of alkali solutions with dolomite crystals.

## 2. Materials and Methods

### 2.1. Materials

**Figure 1:**
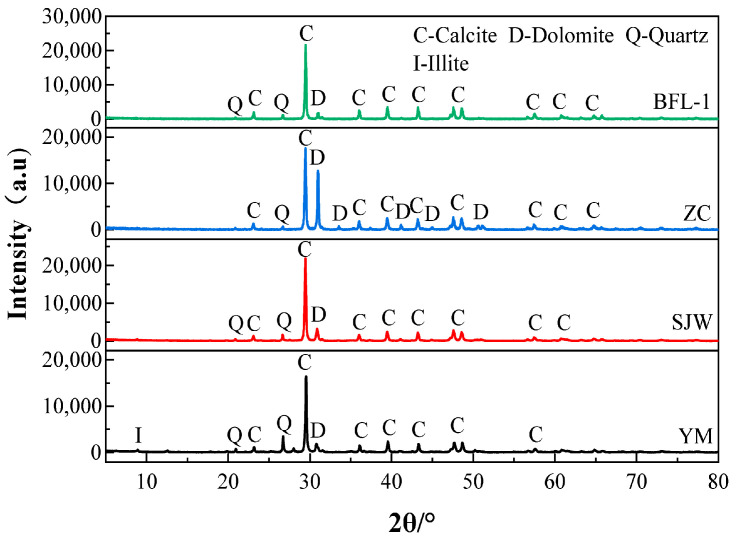
XRD patterns of rocks BFL-1, ZC, YM, and SJW.

This paper engaged dolomitic limestone BFL-1, ZC, YM, and SJW. BFL-1 was obtained from Shandong Province, China, ZC was obtained from Hebei Province, China and YM and SJW were obtained from Guizhou Province, China. Dolomitic limestones BFL-1 and ZC are classified as marine carbonate rocks and belong to the Cambrian system. After the end of the sedimentary period, an irregular dolomitization gave rise to a leopard-like structure. Dolomitic limestones YM and SJW belong to the Ordovician carbonate rocks, which are composed of light gray and gray microcrystalline fine-grained dolomites and limestones, and limestones and argillaceous dolomites build a layered structure with each other. Table 1 provides the chemical composition of dolomitic limestone. The results of MgO in dolomitic limestone BCL-1, ZC, YM, and SJW are 1.19%, 6.65%, 2.48%, and 2.55%, respectively. Figure 1 is the X-ray diffraction pattern of dolomitic limestone, determined in accordance with JY/T 0587-2020 [33]. The results indicate that dolomitic limestones BFL-1, ZC, YM, and SJW are principally composed of calcite, dolomite, and quartz crystals. YM also contains a small portion of illite crystals. The content of dolomite in dolomitic limestone was assessed using the XRD–Rietveld method. The amount of dolomite in dolomitic limestone BFL-1, ZC, YM, and SJW was 8.6%, 26.8%, 6.9%, and 16%, respectively.

**Figure 2:**
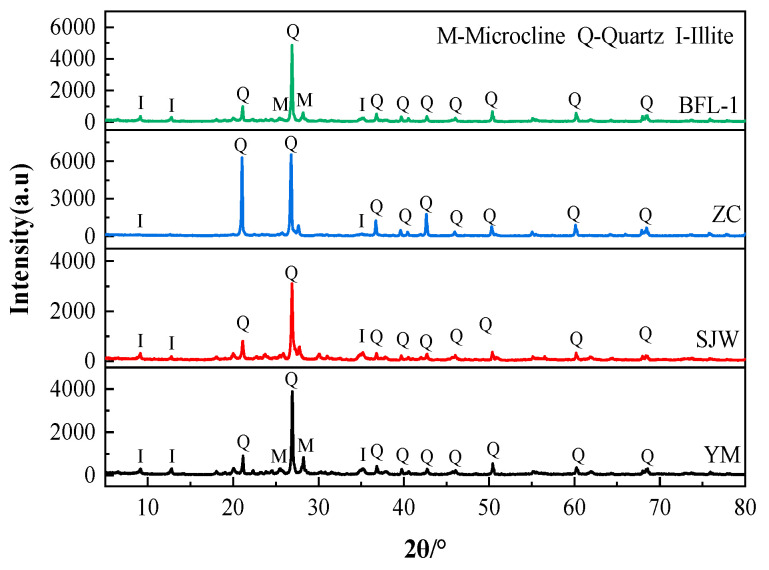
XRD patterns of acid-insoluble residues of dolomitic limestone.

Dolomitic limestone samples were selected and ground to pass a sieve size of 0.08 mm. A 30 g sample of the ground powder was weighed. The powder was added to a glass beaker and submerged in a constant temperature water bath of 60 °C. 3 hydrochloric acid was slowly added, drop by drop, while keeping stirring until evidence of bubbling dissipated, indicating that dissolution was complete (The hydrochloric acid reagent used in this paper was sourced from Xilong Science Co., Ltd., located in Shantou, Guangdong Province, China). The solution was then filtered and the residue removed from the filter and dried in an oven at 60 °C for 24 h. The acid-insoluble substances in dolomitic limestone BFL-1, ZC, YM, and SJW were 4.22%, 3.50%, 21.75%, and 9.56%, respectively. Figure 2 shows the X-ray diffraction pattern of acid-insoluble substances in the rocks. From interpreting the XRD results we can see the acid-insoluble substances of dolomitic limestone YM and SJW were primarily quartz and illite. The dolomitic limestone YM also contained a small amount of microcline and albite. The acid-insoluble substances of dolomitic limestone BFL-1 and ZC were primarily quartz, with only a small amount of illite present. The results of quantitative analysis of each of the minerals of the acid-insoluble substances of rocks using the XRD–Rietveld method is shown in Table 2. The quartz minerals of dolomitic limestone BFL-1, ZC, YM, and SJW are 2.84%, 2.04%, 7.24%, and 3.50%, respectively. The clay minerals (illite) contents were 0.96%, 0.46%, 9.71%, and 5.38%, respectively.

**Figure 3:**
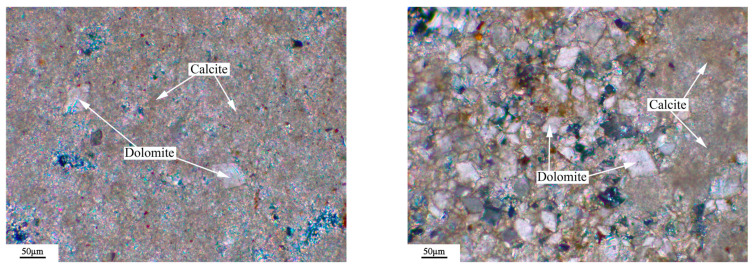
Polarizing microscope images: orthogonal polarizing microscope of BFL-1.

**Figure 4:**
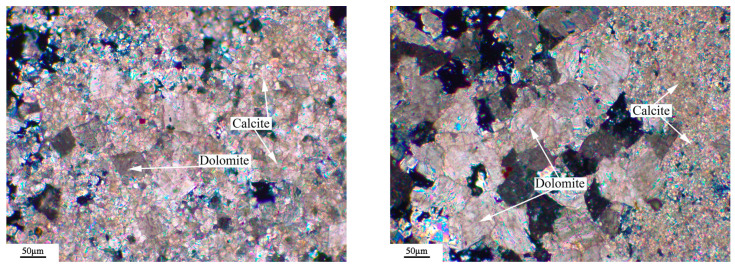
Polarizing microscope images: orthogonal polarizing microscope of ZC.

**Figure 5:**
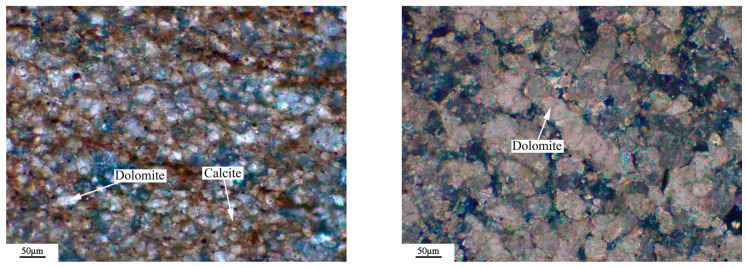
Polarizing microscope images: orthogonal polarizing microscope of YM.

**Figure 6:**
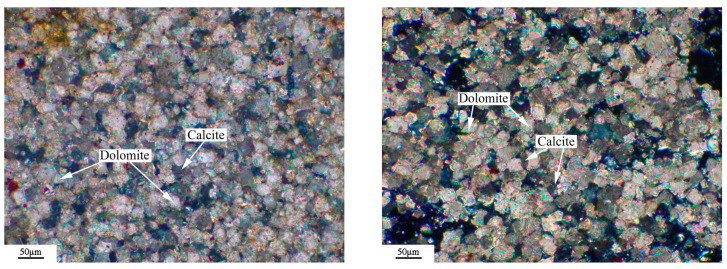
Polarizing microscope images: orthogonal polarizing microscope of SJW.

The microstructure of the rock was observed by a Leica DM750P polarizing microscope (Leica Microsystems, Shanghai, China), and the results were shown in Figure 3, Figure 4, Figure 5 and Figure 6. Figure 3, Figure 4, Figure 5 and Figure 6 show microphotographs obtained from different areas using the same polarizer. The dolomitic limestone BFL-1 was mainly composed of micritic calcite and dolomite with a particle size from 10 to 60 μm. The dolomitic limestone ZC was mainly composed of calcite with a particle size of less than 20 μm and dolomite from 20 to 90 μm. A portion of the dolomite was in a mosaic structure, and the other dolomite was in a dispersed distributed structure. It also had a small amount of microcrystalline quartz. The dolomitic limestone YM was mainly composed of calcite from less than 20 μm and dolomite with a particle size from 10 to 40 μm. The dolomite was mainly presented with a mosaic structure, and the other dolomite was in a dispersed distributed structure. It also contained a small amount of dispersed microcrystalline quartz. The dolomitic limestone SJW was mainly composed of calcite of different particle sizes, and dolomite with a particle size from 10 to 40 μm. The dolomite was mainly presented with a mosaic structure and a small amount distributed in a dispersed distributed structure, containing a small amount of dispersed microcrystalline quartz.

### 2.2. Analytical Methods

#### 2.2.1. Migration Characterization of Na^+^ Ions in Rocks

**Figure 7:**
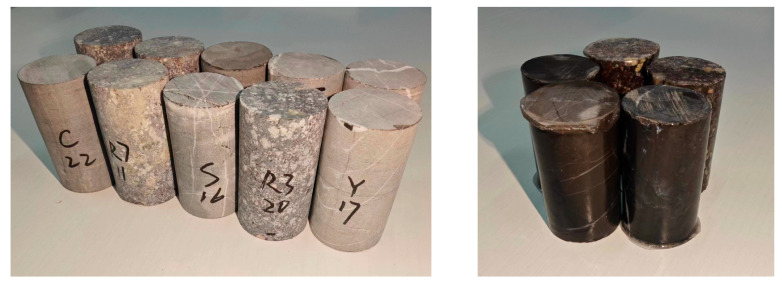
Rock columns used for migration of Na^+^ ions.

This paper aims to examine the migration of ions in rocks. Two approaches were adopted to achieve this goal: a column experiment to investigate ion migration in rock and an experiment to investigate ion migration in rocks of different particle sizes. The paper also characterizes Na^+^ ion migration in rock columns. A core drilling device was used to procure cylindrical rock samples measuring Φ30 × 50 mm. Epoxy resin was uniformly applied onto the perimeter of the rock samples to prevent alkali ion migration into the sample matrix from the sides such that alkali ion could only migrate from the top and bottom surfaces of the sample (see Figure 7). The treated rock samples were immersed in 1 mol/L NaOH at 80 °C with a solid to liquid ratio of 1:5. Following curing for 14, 21, and 35 days the rock samples were removed and, using a slicing machine (Buehler Ltd., Shanghai, China), cut parallel to the top or bottom surface of the rock samples to produce rock slices. The rock slices were produced at 3 mm increments. After accounting for blade thickness of the cut (1.0 mm) the resultant rock slice was 2 mm thick. After each curing period, slices at the 0–2 mm, 3–5 mm, 6–8 mm, 9–11 mm, 12–14 mm, and 15–17 mm positions were cut. All rock slices were ground until they were below 0.080 mm. An amount of 1 g of the powder samples was added to 75 mL of deionized water and soaked for 24 h, then filtered to solid samples. The resultant filtrate was made up to 100 mL in a volumetric flask. The quantity of sodium ions (Na^+^) in the various samples was examined based on the method according to ASTM D1976-20 [34] with a Thermo Fisher ICAP PRO ICP-MS (Thermo Fisher Scientific Inc. Shanghai, China).

Characterization of Na^+^ ion migration in rock particles of different particle sizes was explored. The dolomitic limestone was subjected to crushing using a jaw crusher (Wuxi Jianyi Instrument & Machinery Co., Ltd., Wuxi, China) followed by screening into rock particles of 0.3–0.6 mm, 5–10 mm, and 10–16 mm through a vibrating screen. The rock particles were dried at 105 °C.

The prepared rock particles were immersed in 1 mol/L NaOH solution at 80 °C with a solid–liquid ratio of 1:5. After curing periods of 21, 35, 49, 72, 105, 135, 170, 210, 240, and 300 days, 15 g samples were taken from the rock particles for each particle size. The rock particles were washed with deionized water and dried in an oven at 105 °C for 10 h. The dry rock particles were ground to less than 0.080 mm using a vibration mill (RETSCH GmbH, Shanghai, China). An amount of 1 g of the powder samples was added to 75 mL of deionized water and soaked for 24 h, then filtered to solid samples. The resultant filtrate was made up to 100 mL in a volumetric flask. The quantity of Na^+^ ions in the various samples was also examined using the Thermo Fisher ICAP PRO ICP-MS (Thermo Fisher Scientific Inc. Shanghai, China).

#### 2.2.2. Determination of Degree of Dedolomitization

**Figure 8:**
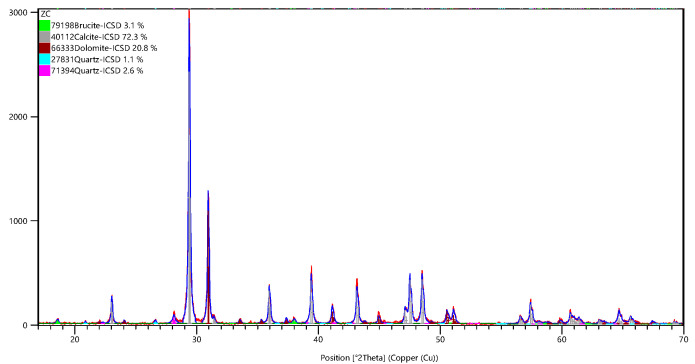
The XRD–Rietveld fitting results for rock sample ZC.

Quantitative determinations of dolomite in each sample were made using the XRD–Rietveld method [33]. The X-ray powder diffraction data were collected at room temperature, using a Rigaku Smart Lab 3 kW diffractometer (Rigaku Corporation, Beijing, China) with a scanning diffraction angle in the range of 17° to 70° at 1°/min. High Score Plus 3.0 software was used to determine the content of minerals in different reacted rock samples, and the degree of dedolomitization was calculated according to the decrease of the content of dolomite. Refinement quality was evaluated visually and through the (Rwp < 10% and GoF < 2). Figure 8 shows the XRD–Rietveld fitting results for rock sample ZC.

#### 2.2.3. Determination of the Pore Structure of Rocks

The investigation of the porosity and pore size distribution of the rock samples was conducted via nitrogen adsorption method (N_2_GA). The samples were analyzed with Gold APP V-Sorb 1800 Surface Area and Pore Size Analyzer (Gold APP Instruments Corporation Ltd., Beijing, China). A total of 5 g of samples with 0.16 mm to 0.32 mm particle size were weighed and applied to the specific surface area and pore size analyzer for determining the pore size distribution and the total pore volume of the rock particles.

#### 2.2.4. Microstructural Analysis

After being immersed in a 1 mol/L NaOH solution at 80 °C for varying lengths of time, dolomitic limestone particles were rinsed with distilled water and dried in an oven at 105 °C for 10 h. Rock particles were stabilized in epoxy then polished using a Buehler Auto Met 250 grinding and polishing system (Buehler Ltd., Shanghai, China). The dolomite components of the rock samples were then analyzed in Zeiss Ultra55 FESEM and BSE (Carl Zeiss AG, Shanghai, China) to provide details of its morphology. The FESEM and BSE imaging was performed at the most optimal imaging conditions of an acceleration voltage of 15 kV, a beam current of 5 nA, and a working distance of 8.5 mm. The depth of dedolomitization of dolomite was determined using the measurement tool function of Photoshop software (Version 21.0.1).

## 3. Results

### 3.1. The Amount of Na^+^ Ion Migration in Rock Columns

**Figure 9:**
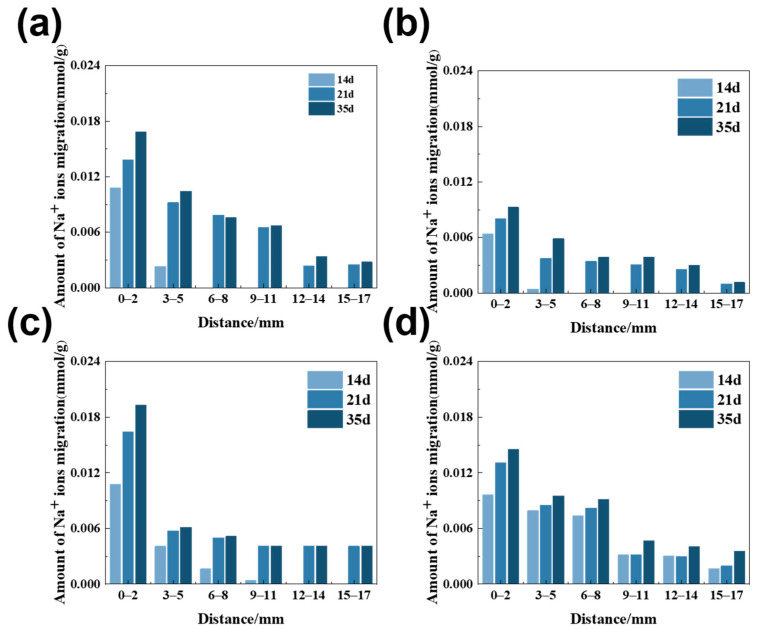
The amount of Na^+^ ion migration in the rock columns of dolomitic limestones in a 1 mol/L NaOH solution at 80 °C ((**a**) BFL-1, (**b**) ZC, (**c**) YM, (**d**) SJW).

Na^+^ ion migration tests were utilized to quantitatively characterize ionic transport behavior of alkali ions in rocks. The amount of Na^+^ ion migration in dolomitic limestone samples immersed in a 1 mol/L NaOH solution at 80 °C for different ages is illustrated in Figure 9. The amount of Na^+^ ion migration discussed here represents the relative changes in Na^+^ ions compared to the reference samples immersed in deionized water, which came from subtracting the initial Na^+^ ion migration values from the reference samples. Due to the non-uniform distribution of mineral composition in the rock, this property will cause the measured Na^+^ ion migration to vary to some extent.

As shown in Figure 9, after the curing age of 14 days, the amount of Na^+^ ion migration in dolomitic limestone BFL-1, ZC, YM, and SJW shows a predominant location from 0 to 5 mm in depth. The amount of migration decreases with depth and tends to stabilize. The amount of Na^+^ ion migration in the middle of the column was relatively small. Over long curing age, Na^+^ ions continued to migrate into the interior of the rock. After curing for 21 days, the amount of Na^+^ ion migration stabilized; within the next observing 14 days of curing, increase in Na^+^ ions migration was not significantly observed.

### 3.2. The Amount of Na^+^ Ion Migration in Rocks of Different Sizes

**Figure 10:**
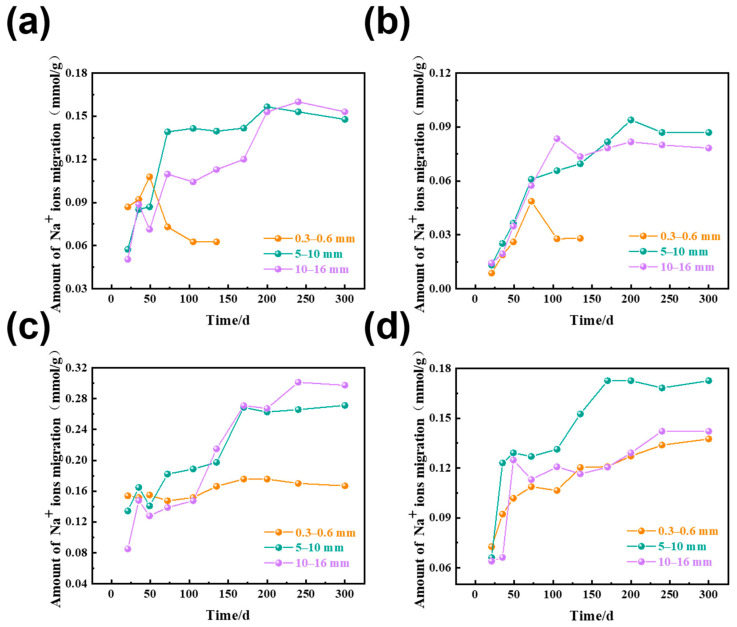
The amount of Na^+^ ion migration in rocks of different sizes of dolomitic limestones in a 1 mol/L NaOH solution at 80 °C ((**a**) BFL-1, (**b**) ZC, (**c**) YM, (**d**) SJW).

The amount of Na^+^ ion migration in dolomitic limestone with particle sizes of 0.3–0.6 mm, 5–10 mm, and 10–16 mm that were immersed in 1 mol/L NaOH solution at 80 °C is shown in Figure 10, in which the amount of Na^+^ ion was calculated by subtracting the initial content of the reference samples immersed in deionized water from the Na^+^ ion content in the cured specimens. There was some fluctuation in the data because of the non-uniform mineral distribution throughout the rock.

As illustrated in Figure 10, during the 72 days of curing, the amount of Na^+^ ion migrated in dolomitic limestone with varying particle sizes increased, although the total amounts of migration were small. The particle size of 5–10 mm had a significantly higher rate of migration than the other sizes. In dolomitic limestone with 0.3–0.6 mm particle sizes of BCL-1 and ZC, the dedolomitization was essentially complete and the rate of ion migration had stabilized after 135 days of curing. Subsequently, there were no additional measurements taken at the further ages of curing. The amount of Na^+^ ion migrated in the larger particle sizes of dolomitic limestone had stabilized in the longer curing ages. At 170 days of curing, the amount of Na^+^ migrated in dolomitic limestone ZC at particle sizes of 5–10 mm and 10–16 mm was 0.082 and 0.078 mmol/g, respectively. At 300 days of curing, the amount of Na^+^ ion migrated in dolomitic limestone ZC at particle sizes of 5–10 mm and 10–16 mm was 0.086 and 0.079 mmol/g, respectively.

Comparing the Na^+^ ion migration of dolomitic limestone with various particle sizes, the amount of Na^+^ ion migration was relatively higher in dolomitic limestone with a particle size of 5–10 mm and lower in dolomitic limestone with a particle size of 0.3–0.6 mm. Na^+^ ion migration in the dolomitic limestone that had a particle size of 10–16 mm was in between these two ranges of dolomitic limestone.

### 3.3. The Amount of Ca^2+^ and Mg^2+^ Ions Dissolution in Rocks of Different Sizes

**Figure 11:**
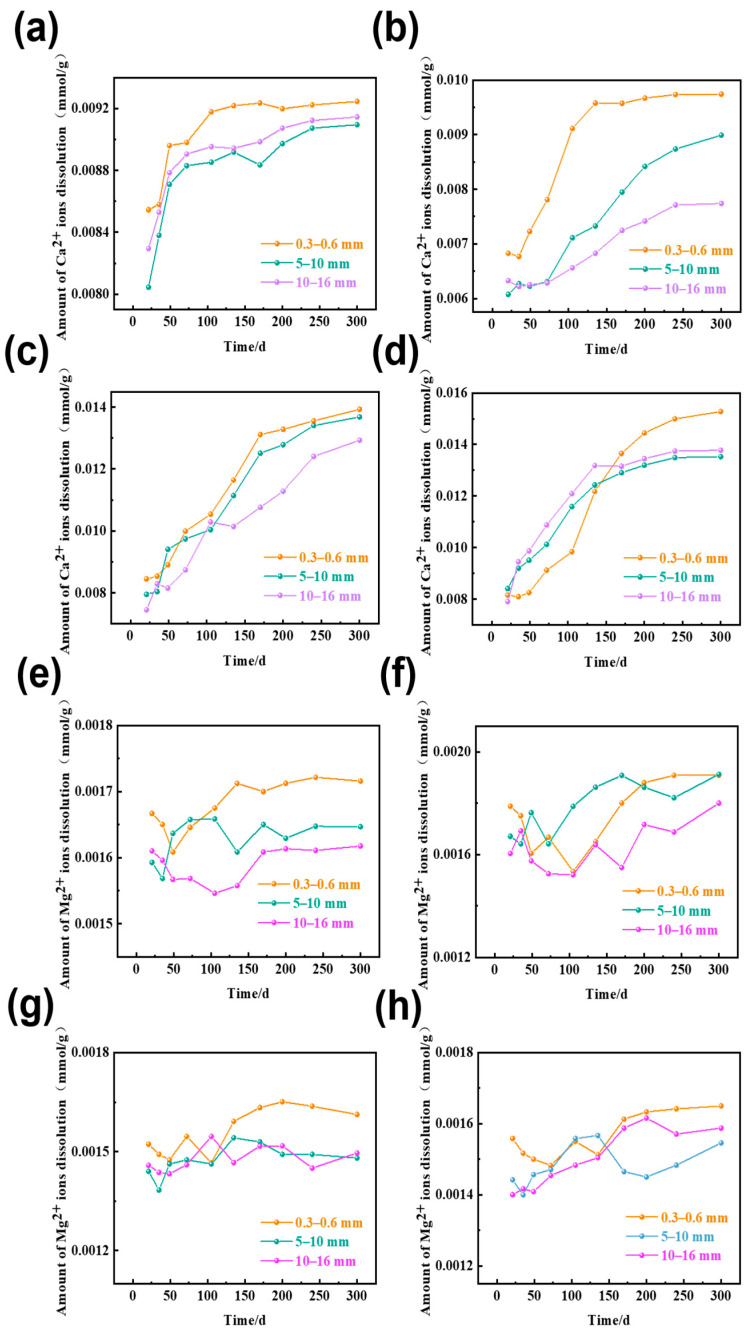
The amount of Ca^2+^ and Mg^2+^ ions dissolution in rocks of different sizes of dolomitic limestones in a 1 mol/L NaOH solution at 80 °C ((**a**) BFL-1- Ca^2+^, (**b**) ZC- Ca^2+^, (**c**) YM- Ca^2+^, (**d**) SJW-Ca^2+^, (**e**) BFL-1- Mg^2+^, (**f**) ZC-Mg^2+^, (**g**) YM-Mg^2+^, (**h**) SJW-Mg^2+^).

To assess the potential effects of calcium and magnesium ions generated by the dissolution of the rocks on sodium ion migration behavior, rock samples were analyzed using ICP-MS to simultaneously measure the concentrations of calcium and magnesium ions. Figure 11 shows the dissolution of Ca^2+^ and Mg^2+^ in dolomitic limestone with particle sizes of 0.3–0.6 mm, 5–10 mm, and 10–16 mm that were immersed in 1 mol/L NaOH solution at 80 °C.

As illustrated in Figure 11, the solubility of calcium ions in NaOH solution is consistently increasing with additional reaction time; however, the overall dissolution remains marginal, with all dissolution values remaining below 0.016 mmol/g. Meanwhile, the solubility of magnesium ions did not vary significantly over time, and magnesium solubility remained low overall, with all dissolution values remaining below 0.0024 mmol/g.

### 3.4. Degree of Dedolomitization

**Figure 12:**
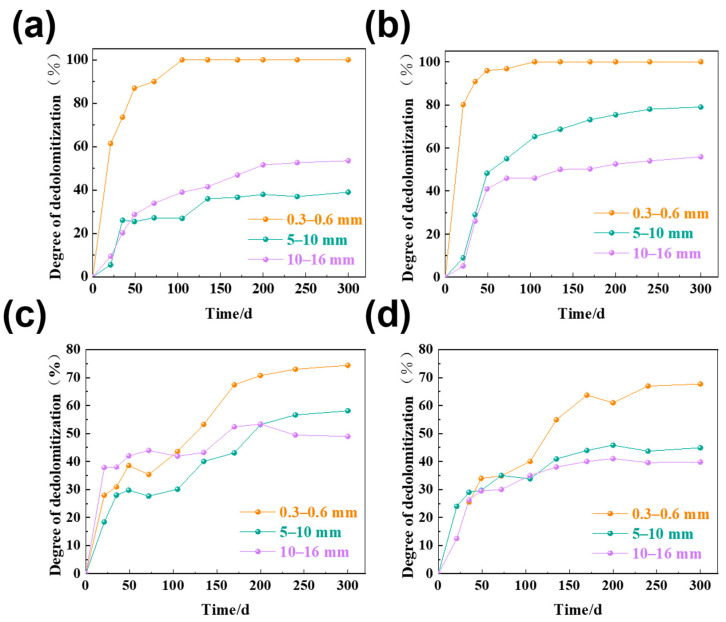
The degree of dedolomitization of different sizes of dolomitic limestones in a 1 mol/L NaOH solution at 80 °C ((**a**) BFL-1, (**b**) ZC, (**c**) YM, (**d**) SJW).

The degree of dedolomitization was evaluated, and it was correlated with the amount of migration of alkali ions in the solution. The degree of dedolomitization in the dolomitic limestone for particle sizes of 0.3–0.6 mm, 5–10 mm, and 10–16 mm that were immersed in a 1 mol/L NaOH solution at 80 °C, as shown in Figure 12. There were some fluctuations in dedolomitization in the testing, which may be a result of the uneven distribution of dolomite in dolomitic limestone.

As illustrated in Figure 12, the degree of dedolomitization in dolomitic limestones of various particle sizes increases with an increase in curing time. The smaller sized dolomitic limestones observed a faster dedolomitization process and greater degree of dedolomitization than the larger particle sizes. Dolomitic limestone BFL-1 (0.3–0.6 mm particle size) showed 100% dedolomitization within 105 days, while the larger particle sizes (5–10 mm and 10–16 mm) showed less than 60% dedolomitization. It can be seen that only dolomitic limestones BFL-1 and ZC (0.3–0.6 mm particle size) reached complete dedolomitization of 100% dedolomitization after 105 days of curing time. However, for the dolomitic limestones SJW and YM with high clay content, some dolomites still did not react even after 300 days of curing for rocks with particle size of 0.3–0.6 mm. With increased curing age, dedolomitization of larger particle sizes tends to become slow. For dolomitic limestone ZC (particle size 5–10 mm), dedolomitization happens to 65% after 105 days of curing and 78% after 300 days of curing. For dolomitic limestone SJW (particle size 5–10 mm), 34% of dedolomitization occurs after 105 days of curing and the degree of dedolomitization reaction could only reach 44% after 300 days of curing. Within the samples, both the total reaction amount of dolomite and the degree of dedolomitization exhibited relatively high levels in dolomitic limestone ZC and relatively low values in dolomitic limestone BFL-1, YM, and SJW. Initial dedolomitization of dolomitic limestone YM and SJW primarily occurred in the first 170 days and then proceeded at a slower rate, which was similarly observed with Na^+^ ion migration. Within 170 days, Na^+^ ions moved into the rock interior, and showed little change after that point in time. Dedolomitization increased with Na^+^ ion migration as there was an increased amount of Na^+^ ions migrating into the rock interior. Hence, the dedolomitization process is also in part affected by the alkali ion migration rate. When alkali ions rapidly migrate into rocks, dedolomitization occurs rapidly.

### 3.5. Pore Structure

**Figure 13:**
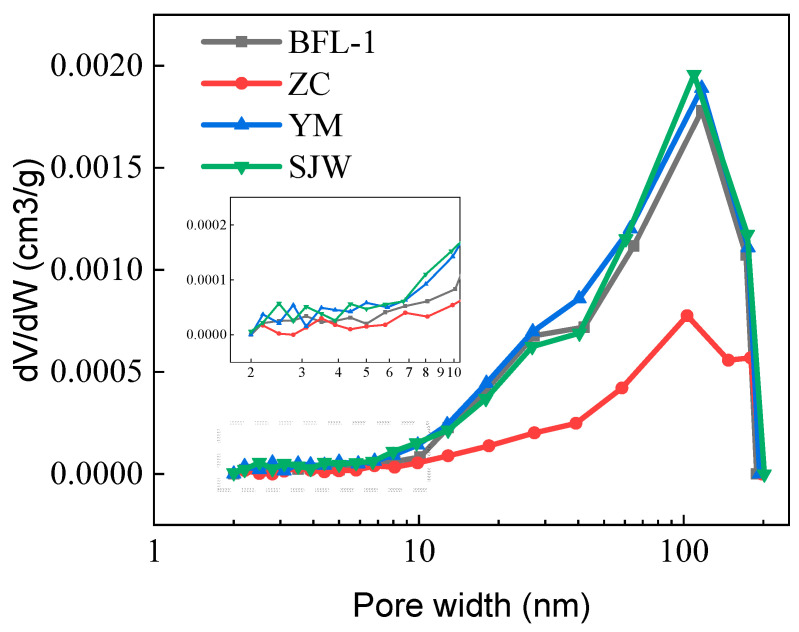
Pore size distribution of rocks BFL-1, ZC, YM, and SJW.

The pore structure properties of rocks affect their transport mechanisms directly and are an important parameter for estimating the migration efficiency of alkali ions in rocks. Figure 13 shows the pore size distribution of rocks BFL-1, ZC, YM, and SJW, which was determined using the BET nitrogen adsorption method. The resultant total pore volume and pore volume for micropores (1–10 nm), mesopores (10–100 nm), and macropores (100–250 nm) is shown in Table 3. Figure 13 indicates that the rocks have a most probable pore diameter of approximately 100 nm. The rocks contain predominantly mesopores and macropores. The total pore volumes of dolomitic limestone BFL-1, ZC, YM, and SJW are 6.4, 3.2, 7.1, and 6.9 mm^3^/g, respectively.

### 3.6. Microstructures of Dedolomitization

**Figure 14:**
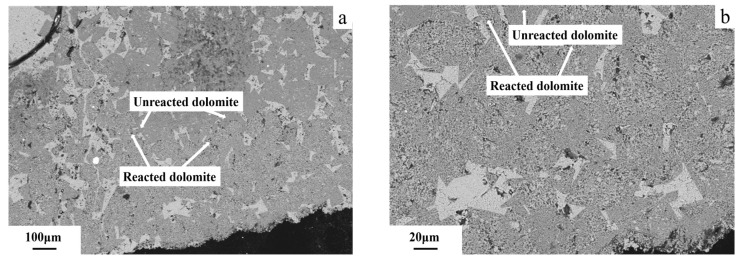
The FESEM images of dolomitic limestone ZC in 1 mol/L NaOH solution at 80 °C for 72 days: (**a**) reacted dolomite at the boundary (bottom zone) and unreacted dolomite in the inner zone (upper zone) and (**b**) magnified reacted dolomite at the boundary.

**Figure 15:**
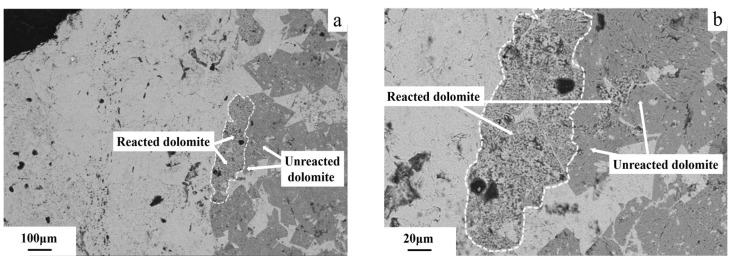
The FESEM images of dolomitic limestone ZC cured in 1 mol/L NaOH solution at 80 °C for 72 days: (**a**) reacted dolomite at the boundary (left zone) and unreacted dolomite in the inner zone (right zone) and (**b**) magnified reacted dolomite at the boundary and unreacted dolomite in the inner zone.

The use of FESEM and BSE to observe the sample surface in detail is a critical step in revealing its microstructure. The images taken using FESEM of 5–10 mm dolomitic limestone ZC as shown in Figure 14 and Figure 15 show results after curing in a 1 mol/L NaOH solution at 80 °C for 72 days. Figure 14a shows a dolomite area displaying a mosaic structure in dolomitic limestone ZC. The dolomite area of approximately 400 μm around the particles shows that some dedolomitization occurs to produce fine products (see Figure 14b), while the internal area of approximately 400 μm distance from the edge of the particle does not exhibit a reaction. The contrast of the reaction zone and unreacted zone area (visible by the white dotted line) has a clear boundary where the two areas meet. Figure 15a shows a matrix that includes calcite and the dolomite area that has a localized mosaic structure within dolomitic limestone ZC. The left area shown on the image is the grayish-white matrix that includes calcite, etc., while the dark gray particle shown on the right is a dolomite crystal. The dolomite crystals located on the left and approximately 800 μm from the edge of the particle show some evidence of dedolomitization producing fine products, while the dolomite area on its right side does not have any visual evidence of the reaction (see Figure 15b). The dedolomitization area is approximately 100 μm wide.

**Figure 16:**
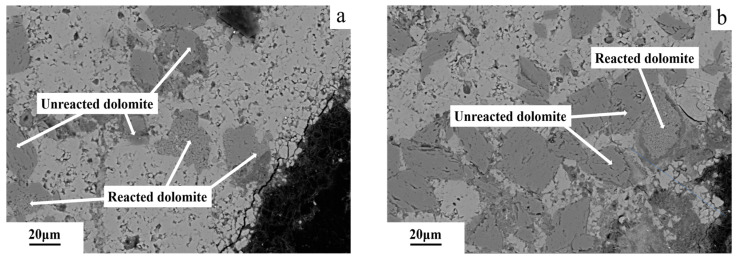
The FESEM images of dolomitic limestone BFL-1 in 5–10 mm cured in 1 mol/L NaOH solution at 80 °C for 72 days: (**a**) dolomite which distributed in a dispersed manner, (**b**) dolomite which had a localized mosaic structure.

The FESEM image of the 5–10 mm dolomitic limestone BFL-1, after curing 1 mol/L NaOH solution at 80 °C for 72 days is shown in Figure 16. The dolomite in the dolomitic limestone BFL-1 is distributed in a dispersed manner. The dolomite within a distance of approximately 110 μm from the particle edge of the dolomitic limestone BFL-1 has been observed to dedolomitization, while the dolomite within the interior region of the dolomitic limestone has basically not reacted. The two dolomite crystals observed at the edge of the dolomitic limestone BFL-1 reveal that one of the dolomite crystals is undergoing dedolomitization, whereas the other side of the crystal remains unreacted (Figure 16a shows these dolomite crystals). In the dolomitic limestone BFL-1, Figure 16b shows the dolomite area that has a localized mosaic structure. Similar to the dolomite crystals noted previously in the dolomitic limestone BFL-1, the dolomite crystals within 80 μm from the edge of the dolomitic limestone BFL-1 have undergone dedolomitization, while the dolomite internal to the particle has remained non-reactive.

**Figure 17:**
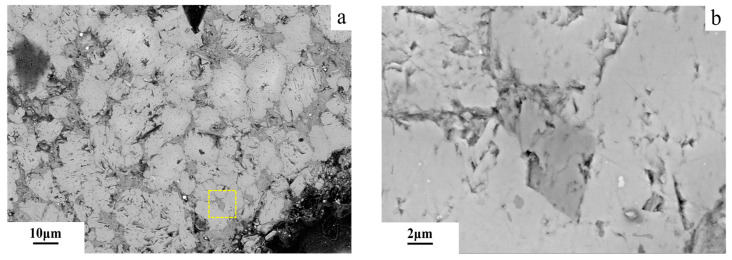
The FESEM images of dolomitic limestone YM in 5–10 mm cured in 1 mol/L NaOH solution at 80 °C for 72 days: (**a**) dolomite at the boundary, (**b**) magnified dolomite at the boundary.

The FESEM image of the dolomitic limestone YM with diameters of 5–10 mm after being cured in an 80 °C and 1 mol/L NaOH solution for 72 days is shown in Figure 17. Figure 17b presents a magnified view of the region outlined by the yellow rectangle in Figure 17a.The dolomite within the dolomitic limestone YM is distributed in a dispersed manner. The dolomite encircled by clay minerals within 50 μm from the edge of the particle has not undergone a visible dedolomitization.

**Figure 18:**
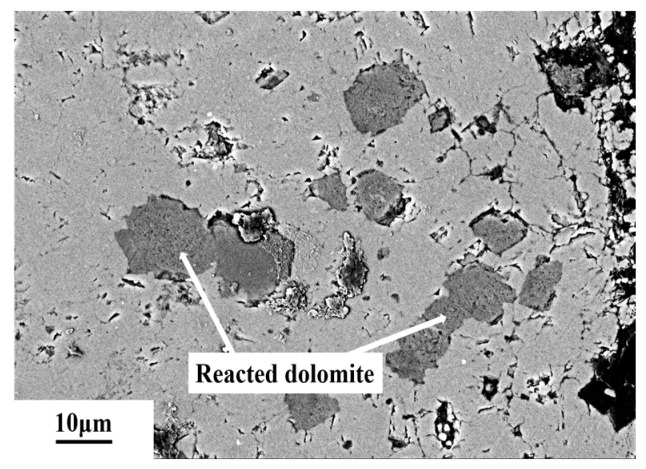
The BSE images of dolomitic limestone SJW in 5–10 mm cured in 1 mol/L NaOH solution at 80 °C for 72 days.

The BSE images of 5–10 mm dolomitic limestone SJW with diameters of 5–10 mm after being cured in an 80 °C and 1 mol/L NaOH solution for 72 days is shown in Figure 18. The dolomite in this rock occurs as fine particles, and most of the dolomite has a mosaic structure. The dolomite particles within a distance of 80 μm from the edges of the particles have undergone dedolomitization.

**Figure 19:**
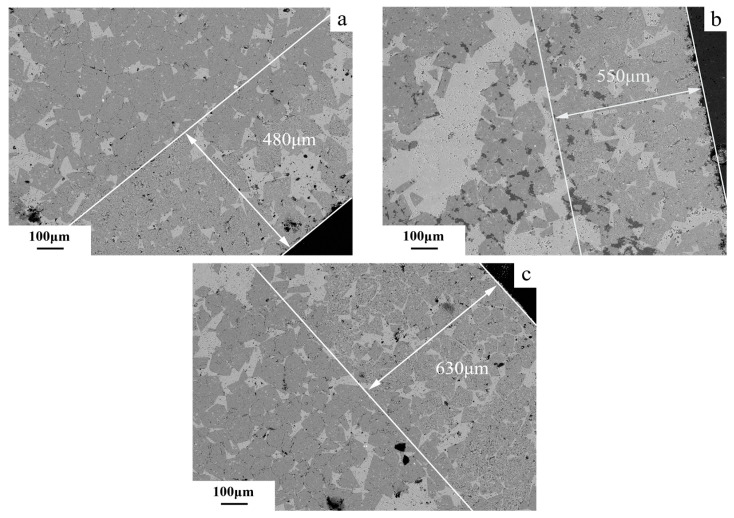
The BSE images of the boundary zone in rock ZC cured in 1 mol/L NaOH solution at 80 °C for different ages (**a**) 49 d, (**b**) 72 d, (**c**) 105 d.

**Figure 20:**
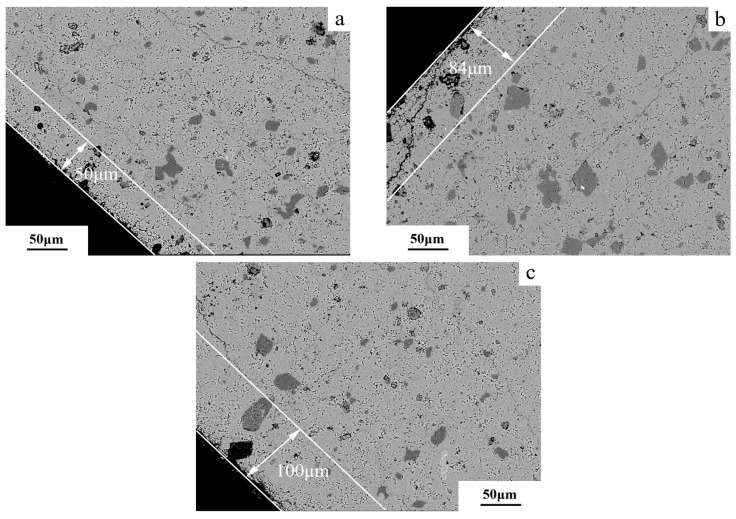
The BSE images of the boundary zone in rock BFL-1 cured in 1 mol/L NaOH solution at 80 °C for different ages (**a**) 49 d, (**b**) 72 d, (**c**) 105 d.

The BSE images of the edge regions of dolomitic limestone samples ZC and BCL-1, respectively, after curing in 1 mol/L NaOH solution at 80 °C for different times are shown in Figure 19 and Figure 20. It is illustrated that after 49 days of curing, the dolomite at a proximity of 480 μm from the edge of the rock displayed dedolomitization, while the dolomite crystals appeared in fine-grain form (Figure 19a). At 72 days, the dedolomitization depth for dolomitic limestone ZC reached 550 μm (Figure 19b); at 105 days, the dedolomitization depth for dolomitic limestone ZC reached 630 μm (Figure 19c). The dolomite closest to the particle edge undergoes dedolomitization first. After the dolomite at the edge has entirely reacted, the dolomite within can begin to react. In this way, dedolomitization has gradually progressed into rock. As shown in Figure 20a, the dolomite at a distance of 50 microns from the edge of rock exhibited the start of dedolomitization after 49 days of curing. At 72 days, the depth of dedolomitization reached 84 μm (Figure 20b); at 105 days, the depth of dedolomitization reached 100 μm (Figure 20c). Similar to the previous result for dolomitic limestone ZC, dedolomitization progresses layer by layer into rock. After the dolomite at the edge has entirely reacted, the dolomite within can begin to react.

## 4. Discussion

This paper aims to examine the alkali ion migration mechanism in dolomitic limestone, thereby impacting dedolomitization. Accordingly, the purpose of the alkali discussion in this paper is to account for the findings based on these factors.

### 4.1. The Influence of Ca^2+^ and Mg^2+^ Ions Dissolution on Na^+^ Ion Migration

When concentrations of solutions are less than 1 mol/L, the solubility of calcite is observed to increase with increasing concentrations of NaOH solution [35,36]. With time, NaOH gradually migrates into the interior of the rock matrix, increasing the concentration of alkaline species, promoting dissolution of calcite. As a result, Ca^2+^ ion leaching rates show an increasing trend with time. On the contrary, brucite solubility in alkaline solutions is considered very low, and dissolution is insensitive to alkaline concentrations [37]. Therefore, dissolution amount of Mg^2+^ ion remains low throughout the reaction time. As shown in Figure 10 and Figure 11, while Ca^2+^ ion can be dissolved to a certain extent, the dissolution amount for Ca^2+^ ion is lower than Na^+^ ion by one order of magnitude to more than ten times and for Mg^2+^ ion it will be two orders of magnitude or tens of times lower than Na^+^ ions. Therefore, it is unlikely that Ca^2+^ and Mg^2+^ ions will significantly affect the diffusion behavior of Na^+^ ions.

### 4.2. The Influence of Particle Size

The dolomitic limestone was cured in a 1 mol/L NaOH solution at 80 °C. Driven by the concentration gradient, Na^+^ ions migrated through the calcite and clay mineral matrix in the rock. There was an upper limit to the migration process. As indicated in Figure 10a,b, after 105 days of curing, the migration of Na^+^ ions reached equilibrium in BFL-1 and ZC where dolomite was completely reacted and the Na^+^ migration was limited. When dolomite crystals were not present, the gaps between calcite crystals provided physical migration paths. The concentration difference of the alkali ions between the curing solution and the pore solution in calcite drove the migration of Na^+^ and OH^−^ into the rock. However, the concentration of alkali ions in the pore solution of calcite was always lower than that of the curing solution.

**Figure 21:**
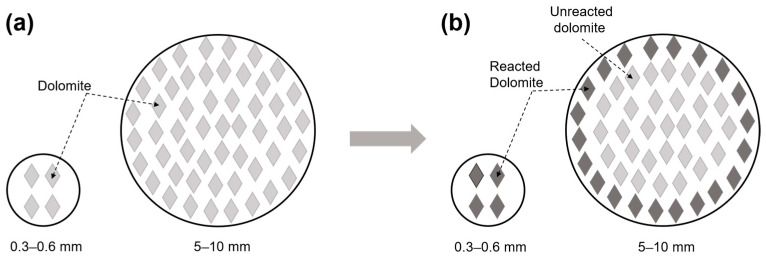
Schematic representation of the dedolomitization of dolomitic limestone with different grain sizes (**a**) before the dedolomitization, (**b**) after the dedolomitization.

For samples with dolomite crystals, when Na^+^ ions migrate to the dolomite interface, dedolomitization occurs. This dedolomitization leads to the production of water-soluble Na_2_CO_3_ and consequently consumes OH^−^ ions and pulls additional OH^−^ ions into the reaction area. Meanwhile, Na^+^ ions also enter the reaction zone accordingly. Figure 21 illustrates the dedolomitization mechanism occurring in dolomitic limestones with different particle sizes. In the examples from 0.3 to 0.6 mm sized dolomitic limestones, high dedolomitization occurred with low dolomite content, and therefore the amount of dolomite consumed per particle was low. However, in larger particle sizes the amount of dolomite consumed is greater, leading to a higher Na^+^ ion migration associated with dedolomitization. This explains the lower Na^+^ ion migration in 0.3–0.6 mm sized dolomitic limestones.

Moreover, if the particle size is too large, the matrix impedes ion migration more readily. As shown in Figure 9, during migration experiment with Φ30 × 50 mm rock column, it could be seen that even the 0–3 mm thin slice had a lower Na^+^ ion migration amount that was dozens to hundreds of times lower than Na^+^ ion migration amount in 10–16 mm dolomitic limestone. The size of rocks has a significant influence on alkali ion migration. This could explain the observation that 10–16 mm dolomitic limestone had a lower migration amount than 5–10 mm dolomitic limestone.

### 4.3. The Influence of Rock Pore Structure on Ion Migration

The formation process of small pores is primarily affected by clay minerals. For different rocks, micropore volumes tend to be larger in rocks with more clay minerals. When clay content surpasses 5%, micropore volume exceeds 0.7 mm^3^/g and when clay content is less than 1%, micropore volume is less than 0.4 mm^3^/g. Illite occurs with carbonate minerals (such as calcite and dolomite) in rocks. The dissolution of carbonate minerals can generate secondary pores, and the presence of illite can provide support for these micropores, preventing them from being compressed or plugged. Additionally, illite has a unique layered structure, with a certain amount of space between its layers [38].

Prior to the initiation of dedolomitization, alkali ion migration is primarily dependent on the porosity of the rock [39]. Porosity provides important pathways for alkali ion migration into the sample. Comparative study of different rock types indicates that the higher the clay content of a rock type, the greater the amount of Na^+^ migrating into the rock. For example, dolomitic limestones containing less clay (ZC and BFL-1) only had Na^+^ ions migrate into a depth of 3–5 mm after curing for 14 days, as shown in Figure 10. Meanwhile, dolomitic limestones containing more clay (YM and SJW) allowed Na^+^ ions to migrate into a depth of 9–11 mm. In this early stage of ion migration, the migration of alkali ions is predominantly controlled by the pores between the crystals; therefore, the greater the porosity of the rock, the faster the alkali ion migration rate. Conversely, in rocks with lower clay content, due to their relatively low porosity, the amount of ion migration is also correspondingly lower.

### 4.4. Alkali Ion Migration After Dedolomitization

In the case of the alkali–aggregate reaction (ASR), alkali ions will migrate away from regions of high cement paste concentrations, into the interior of aggregates where there are lower cement paste concentrations. The migration of alkali ions to the interior of the aggregate is a diffusion process driven by the concentration gradient of alkali ions in solution [7,39]. Likewise, for the reaction removing dolomite to occur, the dolomite must be in contact with alkali ions. The same principle applies, whereby alkali ions will migrate toward the surface of the dolomite crystals, again driven by the concentration gradient. In ADR, OH^−^ ion chemically interact with dolomite and are consumed in the process. This consumption process leads to the further development of a concentration gradient in the reaction system. This concentration gradient promotes migration of OH^−^ ions to the reaction zone, thereby fast tracking dedolomitization. As shown in Figure 14 and Figure 15, after curing in a 1 mol/L NaOH solution at 80 °C for 72 days, dolomite crystals that are 800 μm distant from the edge of dolomitic limestone ZC can react when there are no dolomite crystals at the boundary. This indicates that alkali ions can migrate through the pores into the deeper parts of the rock. When dolomite crystals exist at the boundary, the dolomite that is within about 400 μm of the edge of dolomitic limestone ZC particles shows dedolomitization while the internal dolomite has not undergone the reaction yet. Thus, this shows that dedolomitization limits ion migration into the interior of the rock.

As shown in Figure 19, after curing in a 1 mol/L NaOH solution at 80 °C for 105 days, alkali ions migrated into the rock particle, but the reaction depth was limited to 630 μm from the surface. This suggests that alkali ions chiefly react at the surface or near-surface where dolomite exists, rather deep in the rock. As shown in Figure 9, after curing in a 1 mol/L NaOH solution at 80 °C for 35 days, the amounts of Na^+^ ion migration of dolomitic limestone ZC and YM at depths of 0–2 mm reached 0.012 and 0.021 mmol/g, respectively. In contrast, the amounts of Na^+^ ion migration in either sample at depths greater than 6 mm were only able to reach 0.004 and 0.005 mmol/g. The amounts of Na^+^ ion migration are mainly concentrated within 0–2 mm, and the migration amount at a deeper position of 2 mm is relatively small. This is likely related to the fact that OH^−^ ions were preferentially reacting with the dolomite as they migrated, forming a concentration gradient that drew more OH^−^ ions to the reaction zone. This makes it difficult for OH^−^ ions to further penetrate into the interior of the rock. Once the dolomite is completely reacted, the alkali ions are free to migrate into the interior of the rock. Until that time, the reactions occurring within the reaction zone may have inhibited the migration of alkali ions so that the migration of ions was mainly concentrated in the surface layer of rock and the depth of dedolomitization was relatively shallow.

### 4.5. The Migration Mechanism of Alkali Ions

**Figure 22:**
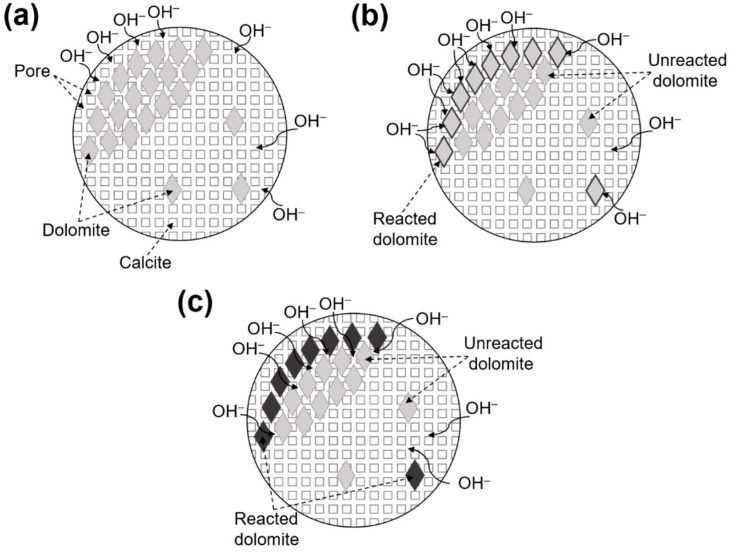
Migration processes of alkali ions (**a**) before the dedolomitization, (**b**) during the dedolomitization, (**c**) after the dedolomitization.

In Figure 22, the process of the migration of alkali ions in dolomitic limestone is illustrated. The dolomitic limestone sample was immersed in a 1 mol/L NaOH solution, and the alkali ions migrated through pores between the crystals, as shown in Figure 22a. During the alkali ions’ migration process through the pores, OH^−^ ions interacted with the dolomite crystals, producing dedolomitization of the dolomite, which consumed a significant amount of OH^−^ ions and therefore reduce the concentration of OH^−^ ions in the reaction area. A concentration gradient is generated between the reaction area and the surrounding environment, which promotes the migration of alkali ions as shown in Figure 22b. Once dedolomitization was complete, alkali ions were no longer confined to the prior state and took the opportunity to migrate further away to deeper areas inside the rock, as shown in Figure 22c.

## 5. Conclusions

This paper evaluated the degree of dedolomitization with respect to the dolomitic limestone samples, BFL, ZC, YM, and SJW, by means of the XRD–Rietveld method. The amount of alkali ion migration was ascertained through ICP-MS. The microstructure and dedolomitization process of dolomitic limestone were examined using FESEM, BSE, and polarizing microscopy. It should be noted that due to the inherent heterogeneity of dolomitic limestone samples studied, the quantitative results presented herein are primarily illustrative. The key value of this work lies in the elucidation of the mechanistic pathways rather than in providing universal quantitative parameters. This paper investigates the migration mechanism of alkali ions in dolomitic limestone. From our experiment work, the following conclusions can be drawn:

1. The pores between crystals are the physical pathways for the migration of alkali cations, whereas the concentration gradient established upon dedolomitization is a driving force for alkali ions migration. The interactions between pores and concentration gradient dictate the migration efficacy of alkali ions.
2. Clay minerals such as illite often have small crystal sizes and between these there is a large number of micropores (1–10 nm). These micropores provide efficient pathways for the migration of alkali ions.
3. The particle size has two effects on alkali ion migration: (a) Dedolomitization facilitates alkali ion migration. The smaller the size, the higher the degree of dedolomitization, but less dolomite content means less alkali ion will migrate after dedolomitization; and (b) The matrix has impediment effect on ion migration. The larger the size of the particle, the stronger the impediment effect.
4. The process of the migration of alkali ions in dolomitic limestone can be represented as: (a) alkali ions migrate in the pores between crystals and contact with dolomite crystals; (b) OH^−^ ions react with dolomite crystals and dedolomitization consumes OH^−^ ions and creates a concentration gradient; (c) alkali ions migrate to the dolomite reaction zone due to the concentration gradient; and (d) after dolomite crystals have reacted completely, alkali ions continue to migrate deeper into the rock.

## Figures and Tables

**Table 1:** Chemical composition of rocks.

Rock	Chemical Composition/wt%							Loss	Total
	SiO_2_	CaO	MgO	Al_2_O_3_	Fe_2_O_3_	K_2_O	Na_2_O		
BFL-1	3.93	52.34	1.19	0.70	0.25	0.16	0.15	40.57	99.35
ZC	2.01	47.07	6.65	0.85	0.25	0.13	0.12	41.43	98.66
YM	11.18	42.26	2.48	2.80	1.03	0.82	0.55	37.19	96.94
SJW	5.99	45.68	2.55	1.49	0.26	0.63	0.20	40.58	96.55

**Table 2:** The content of minerals in acid-insoluble residues of dolomitic limestones.

Rocks	Mineral Content/wt%								AIR/%
	The Mineral Content in the Acid Insoluble				The Mineral Content in Raw Materials				
	Albite	Illite	Quartz	Microcline	Albite	Illite	Quartz	Microcline	
BFL-1	0.00	22.75	67.30	7.35	0.00	0.96	2.84	0.31	4.22
ZC	0.00	13.14	58.29	0.00	0.00	0.46	2.04	0.00	3.50
YM	13.81	44.64	33.29	8.16	3.00	9.71	7.24	1.77	21.75
SJW	0.00	56.28	36.61	0.00	0.00	5.38	3.50	0.00	9.56

**Table 3:** The total pore volume of the rock and its pore volume by pore size.

No	Sample	Clay Minerals Contents/%	Total Pore Volume/(mm^3^/g)	Micropore (1–10 nm) Volume/(mm^3^/g)	Mesopore (10–100 nm) Volume/(mm^3^/g)	Macropore (100–250 nm) Volume/(mm^3^/g)
1	BFL-1	0.96	6.4	0.4	4.9	1.1
2	ZC	0.46	3.2	0.3	1.8	1.1
3	YM	10.04	7.1	0.7	5.3	1.1
4	SJW	5.42	6.9	0.7	5.0	1.2

## Data Availability

The original contributions presented in this study are included in the article. Further inquiries can be directed to the corresponding authors.
